# Hemodynamic characteristics at rupture sites of middle cerebral artery aneurysms: a CFD study with intraoperative correlation

**DOI:** 10.3389/fneur.2026.1984962

**Published:** 2026-10-28

**Authors:** Mingmei Zhao, Shaoyong Yuan, Yaogang Chen, Xiaotong Wang

**Affiliations:** 1 Qingdao Central Hospital, University of Health and Rehabilitation Sciences, Qingdao, China; 2 Qingdao University Affiliated Qingdao Third People’s Hospital, Qingdao, China

**Keywords:** computational fluid dynamics, hemodynamic processes, intracranial aneurysm, middle cerebral artery, wall shear stress

## Abstract

**Objective:**

To characterise the hemodynamic environment at the intraoperatively confirmed rupture point, relative to the aneurysm sac and parent artery, in middle cerebral artery aneurysms (MCAAs), using CT angiography (CTA)-based computational fluid dynamics (CFD).

**Materials and methods:**

Preoperative CTA data from 15 patients with ruptured MCAAs were reconstructed in three dimensions. Transient CFD simulations were run under rigid-wall and Newtonian-fluid assumptions to compute time-averaged wall shear stress (TAWSS), oscillatory shear index (OSI), pressure, and flow streamlines. Rupture regions were identified intraoperatively. No daughter sac, bleb, or secondary lobulation was identified on the preoperative CTA reconstructions or at surgery. Parameters were compared across the three locations using the Friedman test and pairwise Wilcoxon signed-rank tests with Bonferroni correction (*p* < 0.0167).

**Results:**

TAWSS decreased monotonically from the parent artery (median 9.97 Pa) to the aneurysm sac (4.58 Pa) and then to the rupture point (2.14 Pa; all pairwise *p* ≤ 0.003). OSI was higher at the rupture point than at the aneurysm sac and parent artery (*p* = 0.0003 and 0.0002), with no difference between the latter two (*p* = 0.252). Pressure was higher at the rupture point and the aneurysm sac than at the parent artery (*p* = 0.005 and 0.009) but did not differ between the first two (*p* = 0.027). Friedman tests were significant for all three parameters (TAWSS: *p* < 0.0001; OSI: *p* = 0.0004; pressure: *p* = 0.0011). The rupture region coincided spatially with the low-TAWSS and high-OSI areas of the sac: rupture-point TAWSS was lower than sac TAWSS in 14 of 15 patients, and rupture-point OSI exceeded sac OSI in 13 of 15.

**Conclusion:**

CTA-based CFD resolved region-level differences in TAWSS and OSI between the rupture region, the aneurysm sac, and the parent artery. TAWSS and OSI, but not pressure, differed between the rupture point and the aneurysm sac, indicating that low TAWSS and high OSI rather than elevated pressure characterize the rupture point.

## 1 Introduction

Intracranial aneurysms (IAs) are focal dilations of the cerebral arterial wall that form saccular protrusions. Their rupture causes subarachnoid hemorrhage (SAH), with reported mortality of 33–50% (1), and many survivors are left with severe neurological deficits. The mechanisms of IA formation and rupture therefore remain clinically important. Pathogenesis involves several interacting factors, including genetic predisposition, hemodynamic forces, vessel-wall abnormalities, and acquired risk factors such as hypertension and smoking. Among these, hemodynamic forces are central to aneurysm formation, growth, and rupture (2). Meng et al. argued that the long-standing debate over high versus low wall shear stress (WSS) reflects the pathophysiological complexity of aneurysms and that either extreme can promote growth and rupture (3). Perera et al. reported that OSI independently predicts aneurysm rupture (4).

Computational fluid dynamics (CFD) is now the principal tool for relating intracranial hemodynamics to rupture, but the evidence it has produced varies greatly in spatial resolution. Most large studies compare a whole aneurysm with an unruptured control or with the contralateral vessel, so that hemodynamic indices are averaged over the entire sac (5–7). These whole-aneurysm analyses have given inconsistent results: some report lower wall shear stress (WSS) in ruptured aneurysms, others find no difference in low-WSS area while maximum WSS is higher in ruptured lesions, and still others find that patient-specific inflow boundary conditions are not needed to discriminate rupture status (5, 8, 9). An analysis of 359 aneurysms showed that low WSS and high oscillatory shear index (OSI) characterise the body and central regions of the sac regardless of rupture status, so a neck-to-dome gradient is a generic feature of saccular geometry rather than a marker of rupture (10). This leaves an unresolved question: if the hemodynamic signature of rupture is a local property, whole-sac averaging can mask it, and it must be sought at the rupture point itself.

Few studies have resolved hemodynamics at intraoperatively confirmed rupture points, and they disagree. Fukazawa et al., in 12 ruptured middle cerebral artery (MCA) aneurysms studied with CTA-based CFD and video-confirmed rupture points, reported WSS of 0.29, 2.27 and 8.19 Pa at the rupture point, the dome and the parent artery (11); Omodaka et al. reported low time-averaged WSS (TAWSS) at the rupture point relative to the remaining wall in six MCA aneurysms (12); Kono et al. and Suzuki et al. placed rupture points at pressure maxima and at sites of peak-systolic impingement (13, 14); Li et al. likewise found lower WSS at the rupture point than at the remaining sac (15); and Hejčl et al., in a particle-image-velocimetry-verified CFD study, found divergent patterns in which four of six rupture points lay in a low-WSS, high-OSI vortex and one in a high-WSS flow jet (16). The CFD Rupture Challenge showed that even when the local low-shear environment is simulated correctly, the rupture site cannot be reliably identified from the flow field alone: none of the 43 participating neurosurgeons selected the correct site (17). What is needed is not another whole-aneurysm comparison but a paired, within-patient analysis of the hemodynamic environment at the surgically verified rupture point, referenced to the remaining sac and the parent artery in the same model. The present study does this in a single, anatomically homogeneous subgroup — ruptured MCA aneurysms — and so tests whether a consistent local hemodynamic signature exists at the rupture point once whole-sac averaging is removed.

In this study, three-dimensional (3D) models of intracranial aneurysms were reconstructed from preoperative CT angiography (CTA) data and divided into three regions: the rupture point, the aneurysm sac, and the parent artery. CFD was used to simulate the hemodynamic environment, and the main hemodynamic parameters were compared with intraoperative observations of the rupture point. Characterising the hemodynamic profile at the rupture point may help to refine rupture-risk assessment and treatment planning.

## 2 Materials and methods

### 2.1 Patients

We collected clinical data from 17 patients with ruptured intracranial aneurysms who underwent emergency craniotomy in the Department of Neurosurgery at Qingdao Central Hospital between January 2022 and January 2025. Two patients were excluded because of poor image quality that prevented reliable three-dimensional reconstruction. The remaining 15 patients comprised 6 men and 9 women, aged 49–81 years (mean 57 ± 9 years). The study protocol was approved by the Ethics Committee of the Qingdao Central Hospital, and the requirement for informed consent was waived.

### 2.2 Data acquisition

Preoperative imaging was performed using a TOSHIBA 128-slice CT scanner with a tube current of 250 mA, tube voltage of 120 kV, and slice thickness of 0.5 mm. A total of 90 mL of contrast agent was administered through the antecubital vein at a rate of 3.5 mL/s. CT data were saved in DICOM 3.0 format. All surgeries were performed under a Zeiss KINEVO 900 microscope and were video-recorded for subsequent analysis.

### 2.3 Three-dimensional reconstruction of arterial models

CTA data in DICOM format were imported into MIMICS (Materialise, Belgium). Threshold-based segmentation was applied to extract the aneurysm and the feeding arteries, and the models were trimmed to isolate the relevant vascular structures. The resulting models were imported into 3-Matic (Materialise, Belgium) for smoothing, removal of small branches, and trimming of vessel inlets and outlets, and were exported in STL format.

The rupture point was identified intraoperatively by the operating surgeon by direct visual inspection of the wall breach and the surrounding subarachnoid clot, and was subsequently located on the preoperative CTA reconstruction by the same surgeon together with the study investigators. Subsaccular structures were systematically assessed on both the preoperative CTA reconstructions and the intraoperative findings. No daughter sac, bleb, or secondary lobulation was identified in any of the 15 aneurysms; all cases were therefore single-lobed, smooth-walled saccular aneurysms, and the rupture point did not arise from a pre-existing bleb in any case. Because small blebs may be obscured by the smoothing inherent in 0.5-mm-slice CTA reconstruction, a bleb smaller than the slice thickness could nonetheless be missed. The accuracy with which the surgical rupture point can be transferred to the reconstructed geometry is limited by the CTA slice thickness (0.5 mm), by the manual surface registration between the intraoperative visual field and the three-dimensional model, and by the finite size of the breach itself. Taking these contributions together, the spatial uncertainty of the mapped rupture point is of the order of 1–2 mm. Because the aneurysms in this series were small (median volume 31.4 mm^3^, interquartile range 15.4–50.0 mm^3^), this uncertainty is not negligible relative to the dimension of the sac. It is for this reason that the rupture point was analysed as a region rather than as a point, and that the comparison was made at the level of anatomical regions (rupture region, aneurysm sac, parent artery) rather than by pointwise ranking within the sac.

### 2.4 Mesh generation

The STL files were imported into ANSYS Fluent Meshing for surface and volume meshing. Each model contained one inlet, two or three outlets, and the vessel walls. A poly-hexcore volume mesh with five boundary layers along the vessel walls was generated to resolve the near-wall flow, yielding approximately 6.0 × 10^5^ elements per model. Local refinement was applied at the aneurysm neck, where the surface element size was reduced to 0.01 mm to resolve the steep wall-shear-stress gradients at the ostium, while a substantially coarser element size was retained over the remainder of the aneurysm sac, the parent artery, and the far field; the reported element count of approximately 6.0 × 10^5^ per model refers to the whole computational domain and is therefore consistent with a neck-local minimum element size of 0.01 mm.

### 2.5 Mesh sensitivity analysis

A mesh sensitivity analysis was performed on a representative case to verify that the reported results are independent of mesh resolution. Three meshes were generated with the same poly-hexcore topology, five boundary layers, and the same neck-local refinement strategy, differing only in the prescribed element size: coarse (1.2 × 10^5^ elements; minimum element size 0.02 mm at the aneurysm neck), medium (6.0 × 10^5^ elements; 0.01 mm), and fine (3.0 × 10^6^ elements; 0.005 mm). The tabulated element counts are total counts over the whole domain. Peak systolic pressure is reported as absolute pressure in Pa. Using the fine mesh as the reference, the coarse mesh deviated substantially in TAWSS (43.0%) and OSI (51.8%), indicating that it under-resolved the near-wall flow. By contrast, the medium mesh differed from the fine mesh by only 1.2% in peak systolic pressure, 2.8% in TAWSS, and 2.4% in OSI, all below the 5% convergence threshold. The medium mesh was therefore adopted for all 15 cases to balance computational accuracy against computational cost. The meshes and the corresponding mesh-independence metrics are summarised in Table 1.

**Table 1:** Mesh sensitivity analysis on a representative case.

Mesh	Elements	Min. element size at neck (mm)	Peak systolic pressure (Pa, absolute)	TAWSS (Pa)	OSI
Coarse	1.2 × 10^5^	0.02	101,400 (1.0%)	2.748 (43.0%)	0.03142 (51.8%)
Medium	6.0 × 10^5^	0.01	103,700 (1.2%)	1.868 (2.8%)	0.02119 (2.4%)
Fine	3.0 × 10^6^	0.005	102,420	1.922	0.02070

Values in parentheses are relative differences with respect to the fine mesh. The neck size column reports the minimum surface element size applied to the aneurysm neck by local refinement; element sizes elsewhere in the domain are larger, and the element counts are total counts over the whole domain. Pressures are absolute values in Pa; atmospheric pressure is 101,325 Pa, so the tabulated values correspond to gauge pressures of roughly 0–1.1 kPa within the model.

### 2.6 Boundary condition settings

A velocity inlet and pressure outlets were employed as boundary conditions; all outlets were assigned zero relative pressure, with flow distribution determined by the solution. Because inflow velocities differ among patients, a time–velocity waveform from a representative patient’s middle cerebral artery Doppler ultrasound was selected for uniform application. Two considerations support the use of a single waveform. First, the discrimination of aneurysm rupture status from CFD has been shown to be achievable without patient-specific inflow boundary conditions, so that a representative waveform does not by itself compromise the detection of hemodynamic differences between aneurysms (8). Second, and more directly relevant here, the present comparison is not between patients but within each patient: the rupture point, the aneurysm sac and the parent artery are sampled from the same simulation driven by the same waveform, so that any error introduced by the inflow prescription is shared by all three locations and largely cancels in the paired contrasts. The same symmetry applies to the zero-relative-pressure outlets. Flow division among the branches is determined by the solution rather than prescribed, and the shear indices that form the basis of our conclusions (TAWSS and OSI) depend on the local velocity field and its temporal variation rather than on the absolute pressure level. The zero-pressure outlet therefore constrains the pressure analysis, as discussed in Sections 3.1 and 4.2, but is not expected to have a material effect on the TAWSS and OSI contrasts, which are the primary findings of this study. The Reynolds number (Re) of blood in cerebral arteries is typically 600–700; because this is well below the laminar–turbulent transition threshold of 2,300, the flow was treated as laminar. The Newtonian assumption was adopted, a widely used simplification in intracranial aneurysm CFD (18, 19). Comparative studies have shown that Newtonian and shear-thinning blood models yield broadly similar time- and surface-averaged TAWSS, with maximum relative differences of approximately 7% (18), although larger local differences can occur in low-shear regions such as the aneurysm dome (19). Unlike the inflow waveform, the rheological simplification does not act uniformly across the three locations: the Newtonian model has been reported to overestimate wall shear stress specifically in the low-shear region of the aneurysm dome (19), where the rupture point is situated, so this error is concentrated where the primary contrast is drawn and is not cancelled by the paired design. The potential influence of this assumption, particularly on OSI, is acknowledged as a limitation. It is useful to place the magnitude of the modelling assumptions against the magnitude of the effect being reported. The published sensitivity estimates for the simplifications adopted here are approximately 7% for the Newtonian assumption (18), 20% for the rigid-wall assumption (20), and 5–15% for the shape of the inflow waveform (21). The region-level differences reported in this study are considerably larger than any of these: median TAWSS differed by a factor of about 2.1 between the parent artery and the sac and by a further factor of about 2.1 between the sac and the rupture point, with all pairwise contrasts significant at p ≤ 0.003. The assumptions adopted therefore perturb the absolute values of TAWSS and OSI, as acknowledged below, but they are small relative to the between-region contrasts on which the conclusions rest. This magnitude comparison should nonetheless be interpreted cautiously, because the published sensitivity estimates are largely sac-averaged; the rheology, wall-compliance and resolution effects in particular are concentrated in the low-shear regions of the dome (19, 20, 22), which is precisely where the rupture point lies, so they may be larger relative to the rupture-point-versus-sac contrast than to the sac-versus-parent-artery contrast. The fluid simulation was configured as transient, laminar, and incompressible, modeling blood as a Newtonian fluid (density ρ = 1,056 kg/m^3^, dynamic viscosity μ = 0.0035 Pa·s). The vessel walls were assumed rigid and impermeable; gravity was neglected; and the outlet pressure was set to 0 Pa. It should be noted that with a zero-pressure outlet boundary condition, the luminal pressure is determined principally by the inlet-to-outlet pressure drop required to drive the prescribed flow, so that differences in cycle-averaged pressure between closely apposed intrasaccular sites are expected to be small relative to the absolute pressure level; this constraint is relevant to the interpretation of the pressure results in Section 3.1. A pressure-based solver (SIMPLE algorithm) was employed with a residual convergence criterion of 10^−3^. Based on the Doppler ultrasound recordings, each cardiac cycle lasted 0.8 s, with a time step of 0.008 s and up to 100 iterations per step. To ensure convergence, three full cardiac cycles were simulated, and data from the final cycle were used for analysis. This temporal resolution corresponds to 100 time steps per cardiac cycle, which is lower than the resolution used in high-fidelity simulations, and OSI is known to be the reduced hemodynamic index least robust to the computational solution strategy: in 12 MCA aneurysms simulated with high-resolution and normal-resolution strategies, OSI was the index with by far the weakest agreement between the two (R^2^ = 0.23, against R^2^ > 0.9 for cycle-averaged mean and maximum WSS), and the normal-resolution solutions systematically underestimated most indices (22). The direction of this bias matters for interpretation. Because OSI is the reduced index most sensitive to the solution strategy (22), and because the resolution error is not uniform across the sac but is concentrated in the low-shear regions where directional reversals are most frequent — the region in which the rupture point lies — the direction of any resulting bias cannot be assumed to be conservative. Consistent with this, the OSI values obtained here were uniformly low (median 0.008–0.017 across the three regions, maximum 0.045). The OSI results should therefore be read as indicative of relative differences between sites rather than as accurate absolute estimates, and a formal time-step independence study was not performed; a single halved-time-step calculation on a representative case would be required to establish the sensitivity of OSI to temporal resolution.

### 2.7 Hemodynamic parameters evaluated

The following hemodynamic parameters were evaluated: absolute pressure, time-averaged wall shear stress (TAWSS), and oscillatory shear index (OSI).

### 2.8 Statistical analysis

Statistical analysis was performed using SPSS 26.0 (IBM Corp., Armonk, NY). Normality was assessed with the Shapiro–Wilk test. Pressure at all three locations and TAWSS at the rupture point and aneurysm sac deviated from normality (*p* < 0.05), whereas OSI was normally distributed at all three locations. Given this mixed pattern, non-parametric methods were applied uniformly to all parameters to keep the endpoints comparable, and data are presented as the median (lower quartile, upper quartile). Overall comparisons among the three anatomical locations (rupture point, aneurysm sac, parent artery) within the same patient were performed using the Friedman test, with Kendall’s W reported as the effect size for the overall comparison. When the overall test was significant, pairwise comparisons were conducted using the Wilcoxon signed-rank test with Bonferroni correction to control for Type I error inflation due to multiple comparisons; the adjusted significance threshold was set at α′ = 0.05/3 = 0.0167, below which differences were considered statistically significant. Two-sided raw (unadjusted) *p* values are reported, with significance judged against this corrected threshold. For each pairwise contrast, an effect size *r* = |z|/√n was calculated, where z is the tie-corrected normal approximation statistic of the Wilcoxon test and n is the number of paired observations (n = 15). For the comparison of luminal pressure between the rupture point and the aneurysm sac, 9 of 15 patients had identical values at both sites, leaving only 6 informative pairs; this contrast is therefore underpowered and should be interpreted with caution. All tests were two-sided with α = 0.05.

## 3 Results

### 3.1 Pressure comparison

A Friedman test revealed a significant overall difference in pressure among the three locations (χ^2^ = 13.63, *p* = 0.0011, Kendall’s W = 0.454; median absolute pressure 101.9, 101.9 and 101.6 kPa, corresponding to gauge pressures of approximately +0.6, +0.6 and +0.3 kPa relative to atmospheric pressure, at the rupture point, aneurysm sac and parent artery, respectively). Pairwise Wilcoxon signed-rank tests showed that pressure was significantly higher at both the rupture point (*p* = 0.005, *r* = 0.72) and the aneurysm sac (*p* = 0.009, *r* = 0.67) than at the parent artery, whereas no significant difference was found between the rupture point and the aneurysm sac (*p* = 0.027, *r* = 0.57). This comparison is further limited by identical values at both sites in 9 of 15 patients, and the median difference between the rupture point and the aneurysm sac was 0 Pa (rupture point vs. parent artery, +300 Pa, i.e., 0.3%). These small intrasaccular differences should be interpreted in the context of the boundary conditions: with all outlets assigned zero relative pressure, the absolute pressure level is close to atmospheric pressure and the pressure field is governed mainly by the inlet-to-outlet drop required to drive the prescribed flow. Under such a configuration, cycle-averaged pressure is not expected to resolve differences between adjacent sites within a single aneurysm. Luminal pressure therefore distinguished the aneurysm from the parent artery but did not differentiate the rupture point from the aneurysm sac, and the latter observation is as much a property of the pressure boundary condition adopted as of the underlying hemodynamics. These findings are summarized in Table 2 and illustrated in Figure 1a.

**Table 2:** Statistical summary of hemodynamic parameters.

Parameter	Friedman test results (P, W)	RP vs. AN (*p*, *r*)	RP vs. PA (*p*, *r*)	AN vs. PA (*p*, *r*)
Pressure	*p* = 0.0011, W = 0.454	*p* = 0.027, *r* = 0.57 ns	*p* = 0.005, *r* = 0.72*	*p* = 0.009, *r* = 0.67*
TAWSS	*p* < 0.0001, W = 0.813	*p* = 0.003, *r* = 0.73^*^	*p* = 0.0001, *r* = 0.88^*^	*p* = 0.0006, *r* = 0.81^*^
OSI	*p* = 0.0004, W = 0.520	*p* = 0.0003, *r* = 0.84^*^	*p* = 0.0002, *r* = 0.85^*^	*p* = 0.252, *r* = 0.31^ns^

Friedman test with Kendall’s W was used for overall group comparison; pairwise comparisons were performed using Wilcoxon signed-rank tests, and post-hoc effect size r was calculated for inter-group contrasts. (*) Indicates significant difference; ns, non-significant. TAWSS, time-averaged wall shear stress; OSI, oscillatory shear index. RP, AN and PA represent three comparative groups. *p* values are raw (unadjusted); significance for pairwise comparisons was determined against a Bonferroni-corrected threshold of *p* < 0.0167 (0.05/3). Effect size *r* = |z|/sqrt(*n*), where z is the tie-corrected normal approximation statistic of the Wilcoxon signed-rank test and n is the number of paired observations (*n* = 15 for all comparisons). Note that 9 of 15 patients had identical pressure at the rupture point and aneurysm sac; the RP vs. AN pressure comparison therefore rests on only 6 informative pairs and should be interpreted with caution.

**Figure 1:**
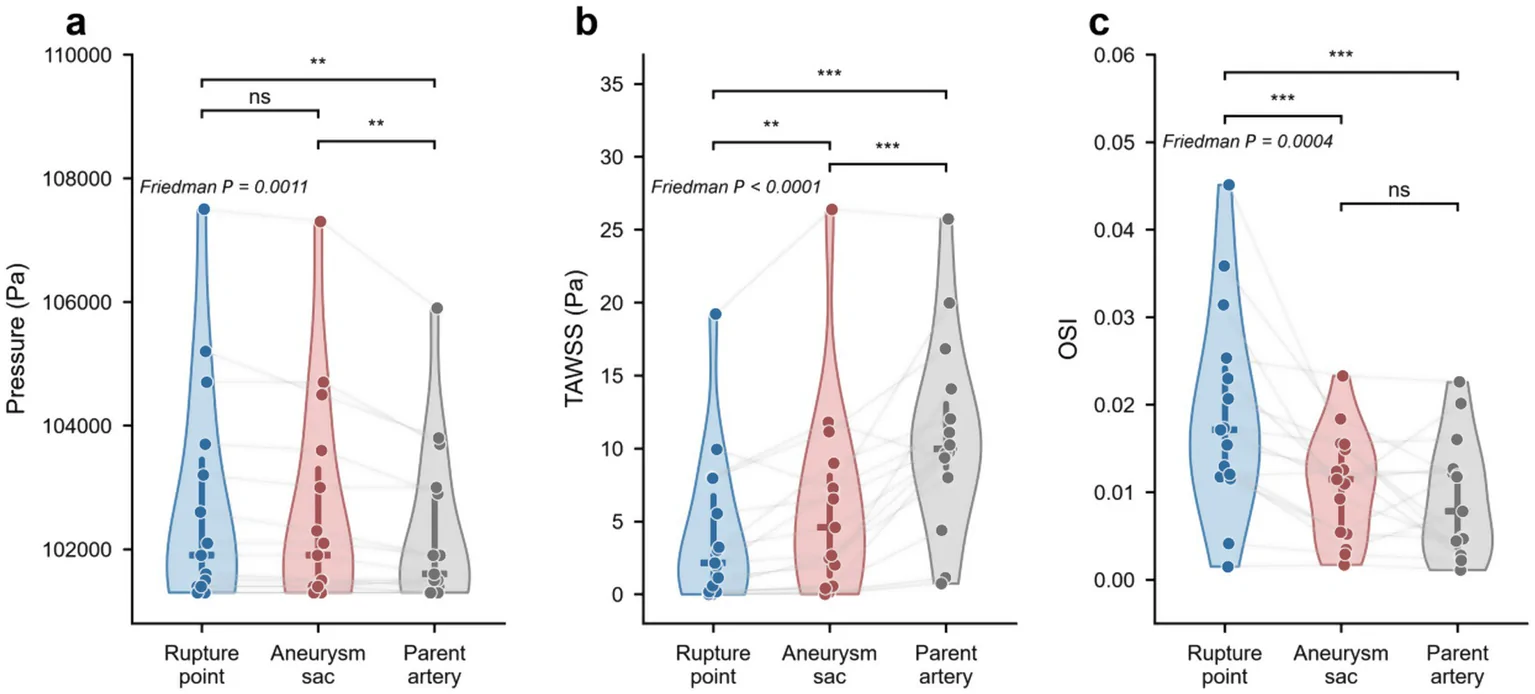
Hemodynamic parameter comparison across the rupture point, aneurysm sac, and parent artery in intracranial aneurysm patients. **(a–c)** Violin plots with overlaid individual data points and paired trajectories comparing **a** luminal pressure, **b** time-averaged wall shear stress (TAWSS), and **c** oscillatory shear index (OSI) at the rupture point (blue), aneurysm sac (red), and parent artery (grey). Each grey line traces a single patient’s measurement across the three anatomical locations. Colored vertical bars denote the interquartile range (IQR); the short horizontal bar within each IQR marks the median. Friedman test *p*-values are shown at the top-left of each panel for overall comparison. Pairwise comparisons were performed using two-sided Wilcoxon signed-rank tests with Bonferroni correction (adjusted significance threshold: *p* < 0.0167); asterisks denote the magnitude of the raw (unadjusted) *p* values (*p < 0.05, **p < 0.01, ***p < 0.001) and ns denotes a comparison not reaching this threshold. TAWSS exhibited a significant monotonic decline from the parent artery toward the rupture point (all pairwise comparisons, *p* < 0.0167), indicating a locally disturbed flow environment at the rupture-prone region. OSI was significantly higher at the rupture point than at both the aneurysm sac and the parent artery (RP vs. AN: *p* = 0.0003; RP vs. PA: *p* = 0.0002), whereas no significant difference was found between the aneurysm sac and the parent artery (*p* = 0.252). Luminal pressure showed significant differences between the parent artery and the other two sites, but not between the rupture point and aneurysm sac after correction. *n* = 15 patients.

A representative case illustrates the aneurysm morphology and the intraoperative confirmation of the rupture point (Figure 2), as well as the simulated pressure, flow streamline, TAWSS, and OSI distributions (Figure 3).

**Figure 2:**
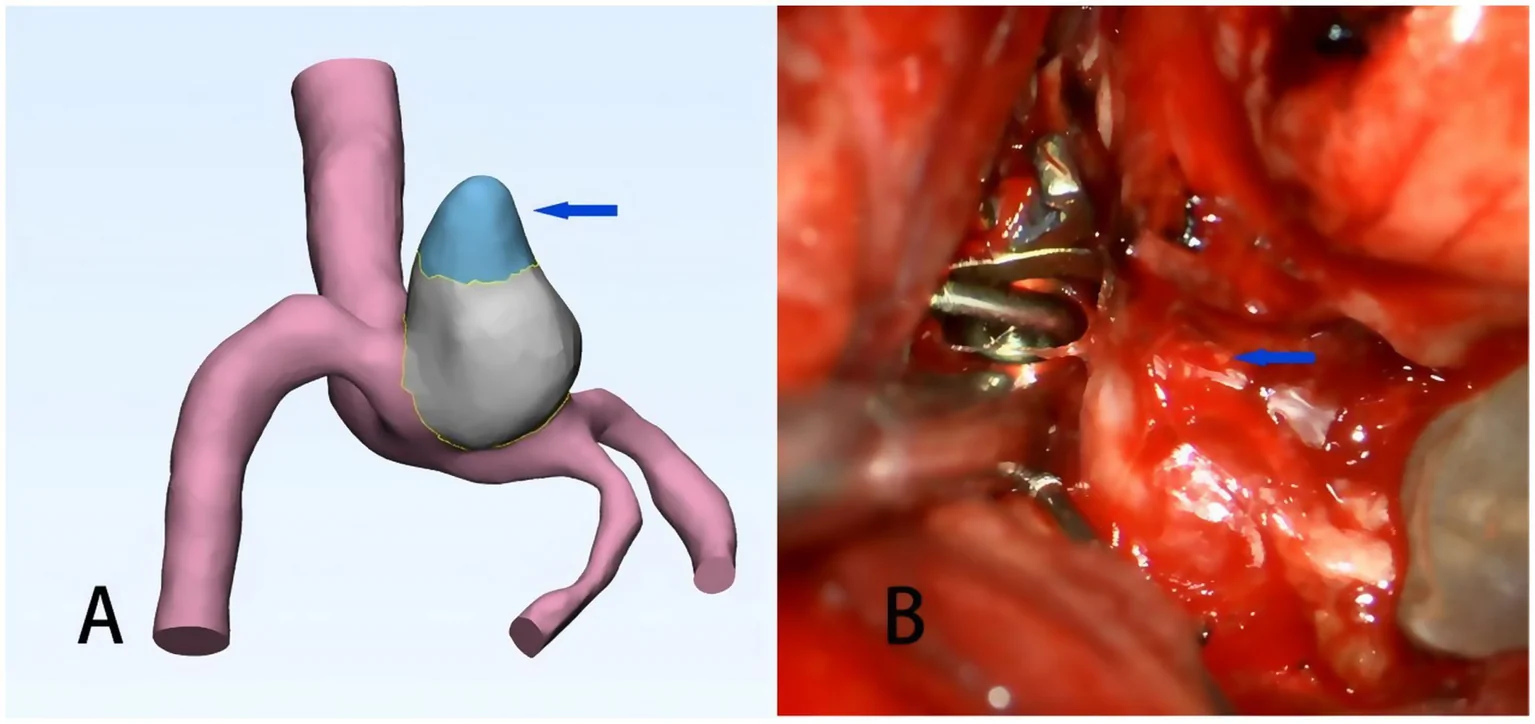
Intraoperative identification of the rupture point. **(A)** Aneurysm morphology on the preoperative CTA reconstruction. **(B)** Intraoperative surgical photograph; the arrow indicates the rupture point as identified by the operating surgeon.

**Figure 3:**
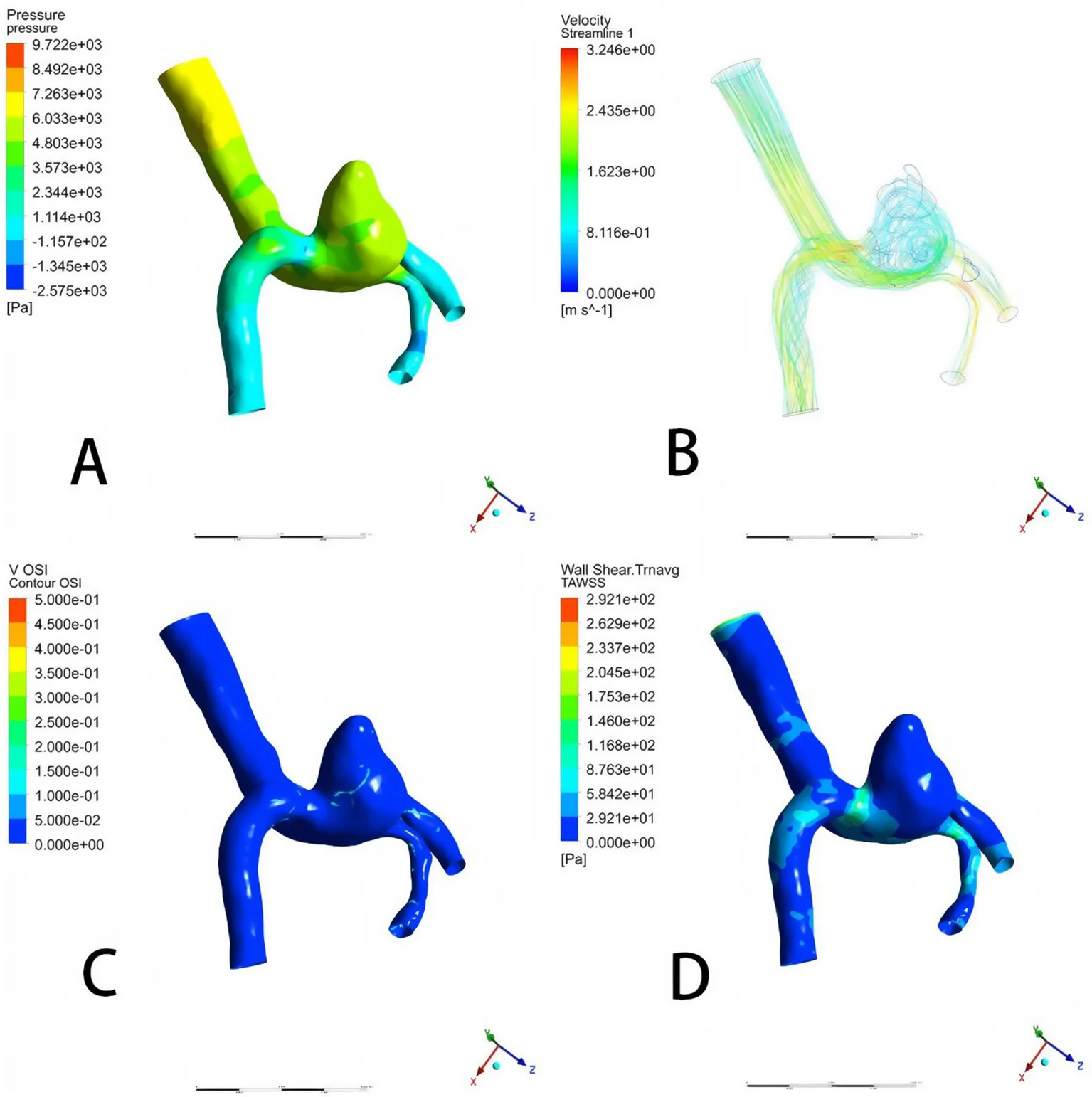
**(A)** Pressure; **(B)** Flow streamline diagram; **(C)** Oscillatory shear index; **(D)** Wall shear stress. Panels **A** and **D** share the same color scale convention: for pressure **(A)**, values are gauge pressures relative to the outlet, which was assigned zero relative pressure, so that the color scale includes slightly negative values in regions where the local pressure falls below the outlet reference; for wall shear stress **(D)**, the color scale denotes instantaneous values at peak systole, whereas the values reported in the text and in Figure 1B are time-averaged over the cardiac cycle (TAWSS), which are therefore roughly an order of magnitude lower than the instantaneous peak values shown here.

### 3.2 Time-averaged wall shear stress (TAWSS)

TAWSS was obtained by integrating the wall shear stress at each nodal point over one cardiac cycle. On the TAWSS contour maps, red regions denote higher shear stress, whereas blue regions denote lower shear stress. All TAWSS values are reported as absolute time-averaged wall shear stress in pascals, without further normalisation; comparisons between patients are supported by the within-patient paired design, in which each patient serves as their own control. The overall Friedman test demonstrated a significant difference among the groups (*p* < 0.0001, Kendall’s W = 0.813). TAWSS decreased monotonically from the parent artery (median 9.97 Pa) through the aneurysm sac (4.58 Pa) to the rupture point (2.14 Pa). Pairwise analyses showed that TAWSS at the rupture point was significantly lower than that at the aneurysm sac (*p* = 0.003, *r* = 0.73) and at the parent artery (*p* = 0.0001, *r* = 0.88); TAWSS at the aneurysm sac was also significantly lower than at the parent artery (*p* = 0.0006, *r* = 0.81). All three pairwise contrasts were significant, with large effect sizes (*r* = 0.73–0.88). These differences are illustrated in Figure 1b and summarized in Table 2.

### 3.3 Oscillatory shear index (OSI)

The oscillatory shear index was defined as the spatially averaged OSI over the vessel wall segments of interest, including the rupture point, the aneurysm sac, and the parent artery. The Friedman test indicated a significant overall group difference (*p* = 0.0004, Kendall’s W = 0.520; median 0.0171, 0.0115 and 0.0078 at the rupture point, aneurysm sac and parent artery, respectively). Pairwise comparisons revealed that OSI at the rupture point was significantly higher than that at the aneurysm sac (*p* = 0.0003, *r* = 0.84) and at the parent artery (*p* = 0.0002, *r* = 0.85), whereas no significant difference was observed between the aneurysm sac and the parent artery (*p* = 0.252, *r* = 0.31). These results indicate that the rupture point is characterized by markedly elevated oscillatory shear, while the aneurysm sac and parent artery exhibit comparable OSI levels. These findings are illustrated in Figure 1c and summarized in Table 2. At the individual level, TAWSS at the rupture point was lower than that at the aneurysm sac in 14 of 15 patients, and sac TAWSS was lower than parent-artery TAWSS in 13 of 15; OSI at the rupture point exceeded the sac value in 13 of 15 patients and the parent-artery value in 14 of 15. The consistency of these contrasts across the cohort indicates that the region-level differences are not driven by a small number of outlying cases.

## 4 Discussion

Middle cerebral artery aneurysms (MCAAs) are among the most common intracranial aneurysms and arise mainly at M1 or M2 bifurcations. Their rupture risk depends on both morphology and hemodynamics, which are coupled: geometry dictates intra-aneurysmal flow patterns, while hemodynamic forces drive initiation, growth, and remodelling (23). In this study we combined CTA-based CFD parameters (WSS, TAWSS, OSI, and pressure) to characterise rupture-related features of MCAAs. Large prospective cohorts, including the International Study of Unruptured Intracranial Aneurysms (ISUIA) (24) and the Unruptured Cerebral Aneurysm Study (UCAS) in Japan (25), provide the main evidence on the natural history and rupture risk of these lesions.

### 4.1 Morphological indices and their limitations

Morphological features are important determinants of rupture risk, and aspect ratio (depth/neck width) was among the first indices proposed; higher values indicate a more elongated dome and, conventionally, a greater likelihood of rupture. Several caveats apply. Aspect ratio is derived from dimensions measured on angiographic or CTA reconstructions, and its discriminative performance varies with location and size, being weaker for MCA aneurysms and for small aneurysms than in pooled cohorts (26, 27). Morphology was not the focus of the present study, although the relevant measurements were available for all 15 patients; the analysis is therefore limited to a within-aneurysm, region-level comparison of hemodynamic parameters, and no morphological index is used here to stratify or validate rupture risk.

Other morphological indices, such as the ellipticity and nonsphericity indices, have also been associated with rupture (4), although they were not evaluated systematically in this cohort. The PHASES score combines patient demographics, clinical history, and aneurysm characteristics and is the current clinical standard for rupture-risk estimation (28).

### 4.2 Relationship between hemodynamic parameters and aneurysm rupture

CFD has proved valuable for analysing intracranial aneurysm hemodynamics. Patient-specific inflow boundary conditions may not be strictly necessary for accurate simulation, which simplifies the modelling and reduces computational cost (8). Cebral et al. established a widely used classification of intra-aneurysmal flow patterns into four types based on flow complexity and stability, which has guided much subsequent work in the field (5).

Wall shear stress (WSS), the tangential force that flowing blood exerts on the vessel wall, is a central factor in intracranial aneurysm pathogenesis, and abnormal WSS is closely associated with formation, growth, and rupture. Excessively high WSS can activate destructive remodelling pathways in endothelial cells: once WSS exceeds a critical threshold, mechanosensation triggers biochemical cascades, including cell proliferation and protease activation, that degrade the elastic lamina, thin the media, and so promote expansion and rupture (23). Low WSS, by contrast, has been linked to atherogenesis and plaque formation and may provoke local inflammation and wall degeneration, increasing rupture risk (6). Zhang et al. reported that extremely low WSS, caused by flow stagnation or intraluminal thrombus, may precipitate local inflammation and lead to rupture (29).

Time-averaged wall shear stress (TAWSS), the average of WSS over a cardiac cycle, decreased monotonically from the parent artery (median 9.97 Pa) to the aneurysm sac (4.58 Pa) and then to the rupture point (2.14 Pa; all pairwise *p* ≤ 0.003; Friedman *p* < 0.0001). This graded decline is consistent with the rupture-prone region lying within a locally disturbed, low-shear environment. At the whole-aneurysm level the evidence is less consistent. In 210 aneurysms, Cebral et al. found no significant difference in the area of low WSS between ruptured and unruptured aneurysms, but did find significantly higher maximum WSS in ruptured aneurysms — the opposite of what we report (5). Such discrepancies may arise because site-specific signals are diluted when the rupture point is not delineated separately. In 238 unruptured aneurysms stratified by PHASES score, however, lower time-averaged WSS was significantly associated with higher predicted rupture risk (9), consistent with our finding that the rupture region lies in a low-shear environment; whole-aneurysm averaging therefore does not necessarily abolish the association when the comparison is between risk strata rather than between ruptured and unruptured groups. In a case–control study of 784 aneurysms matched for location, age, and sex, Chung et al. found that ruptured aneurysms had a lower minimum WSS and a higher mean OSI than stable aneurysms (7), in agreement with our results.

The rupture point was defined intraoperatively by the operating surgeon as an anatomical region of wall breach with surrounding subarachnoid clot, not as a geometrically reproducible coordinate. Comparable studies have addressed the same difficulty by requiring independent review: Fukazawa et al. (11) required agreement among three neurosurgeons, and in the CFD Rupture Challenge none of the 43 participating neurosurgeons identified the correct rupture site (17). Because the position of the rupture point therefore carries an irreducible uncertainty, expressing it as a percentile within a pointwise TAWSS or OSI distribution of the sac would imply a precision the data do not have: a rank computed from an imprecisely localised coordinate cannot be read as a site-specific measurement. We therefore restricted the analysis to region-level comparisons — rupture region versus whole sac versus parent artery — the level of resolution the intraoperative localisation can support. This design still separates the rupture region from the sac without overstating the precision of the localisation.

Although we did not perform a formal within-sac percentile analysis, the absolute values can be compared with previously published within-aneurysm data. Omodaka et al. reported a TAWSS of 1.10 Pa at the rupture point versus 4.96 Pa at the remaining aneurysm wall in six MCA aneurysms with intraoperatively confirmed rupture points (12); the corresponding values in our series were 2.14 Pa and 4.58 Pa. The agreement is close in both magnitude and direction: the sac-wall values agree to within about 8%, and the rupture-point values fall in the same low-shear range. This agreement between two independent cohorts supports the reproducibility of the sac-internal low-shear environment at rupture points, even though neither study can assign the rupture point an exact percentile rank. A third series of 12 ruptured MCA aneurysms with video-confirmed rupture points reported a similar ordinal pattern — mean WSS of 0.29 Pa at the rupture point versus 2.27 Pa at the dome and 8.19 Pa at the parent artery (11) — which, although systematically lower in absolute terms, reproduces both the direction and the parent-artery-to-sac-to-rupture gradient we observed.

One further caveat concerns how the sac-internal low-shear region should be interpreted. Karnam et al., analysing 359 aneurysms, showed that low WSS and high OSI characterise the body and central regions of the sac regardless of rupture status, and that a comparable neck-to-dome gradient is a generic feature of saccular geometry (10). The monotonic decline in TAWSS from parent artery to sac to rupture point is therefore not by itself evidence of a rupture-specific mechanism; it indicates only that the rupture point lies within the sac-internal low-shear region. Because the mapping uncertainty (1–2 mm) is of the same order as the aneurysm dimension, we did not attempt to show that the rupture point occupies a uniquely low-shear location within the sac; the observed pattern (rupture-point TAWSS lower than sac TAWSS in 14 of 15 cases, and sac TAWSS below parent-artery TAWSS in 13 of 15) is accordingly reported as a descriptive consistency rather than as evidence of site specificity. A related question is whether the low TAWSS and high OSI at the rupture point might be a geometric rather than a hemodynamic consequence, since daughter sacs enlarge markedly at rupture (27) and rupture sites are reported to arise predominantly on blebs (30). This does not apply to our cohort: no daughter sac, bleb, or secondary lobulation was identified in any of the 15 aneurysms on the preoperative CTA reconstructions or at surgery, although small blebs below the CTA resolution cannot be excluded. The rupture points therefore lay on an otherwise smooth, single-lobed sac wall, and the focal reduction in TAWSS and elevation in OSI at these sites cannot be attributed to a pre-existing bleb.

The oscillatory shear index (OSI) quantifies directional changes in WSS over a cardiac cycle; a higher OSI indicates greater oscillatory swings in shear stress, which may promote endothelial dysfunction and wall destabilisation. In our cohort, OSI was significantly higher at the rupture point than at the aneurysm sac (*p* = 0.0003) or the parent artery (*p* = 0.0002), with no difference between the sac and the parent artery (*p* = 0.252). Elevated oscillatory shear is therefore a focal feature of the rupture point rather than a global property of the aneurysm-bearing segment, a distinction that whole-aneurysm averaging would obscure. Jiang et al. identified OSI as an independent predictor of rupture in a retrospective study (2). Two aspects of the OSI computation require comment. First, OSI is the reduced index most sensitive to the solution strategy: comparing high- and normal-resolution simulations of 12 MCA aneurysms, only OSI failed to correlate between the two (R^2^ = 0.23), whereas other indices correlated closely (R^2^ > 0.9), and the lower-resolution solutions underestimated most indices (22). Our calculations used 100 time steps per cardiac cycle, and a finer step was not available to confirm these values. Second, and more important for interpretation, the resolution error is not uniform across the sac but is concentrated in the low-shear regions where directional reversals are most frequent, so the direction of any resulting bias cannot be assumed to be conservative. The OSI contrasts between the rupture point and the sac should therefore be interpreted with caution rather than as precise quantitative differences, and a single halved-time-step calculation on a representative case would be needed to establish their sensitivity to temporal resolution.

Luminal pressure behaved differently. Pressure at both the rupture point and the aneurysm sac exceeded that at the parent artery (*p* = 0.005 and *p* = 0.009; Friedman *p* = 0.0011), but the absolute differences were small (median absolute pressure 101.9 vs. 101.6 kPa, that is, gauge pressures of about +0.6 vs. + 0.3 kPa relative to atmospheric pressure), and no significant difference emerged between the rupture point and the sac (*p* = 0.027), a comparison further limited by identical values at both sites in 9 of 15 patients. Thus, while TAWSS and OSI differentiated the rupture point from the sac, pressure did not, indicating that the hemodynamic signature of the rupture point is defined by low shear and high oscillatory shear rather than by elevated static pressure (7). This should not be taken to mean that pressure plays no part in rupture. Studies with precise rupture-site identification report that the rupture site coincides with the pressure maximum or the peak-systolic impingement zone (13, 14); in 23 MCA bifurcation aneurysms, the maximum-pressure area coincided with the rupture point in 5 of 7 ruptured cases, although the normalised pressure difference there was not higher than at thin-walled regions of unruptured aneurysms (13). Our analysis used cycle-averaged luminal pressure and had limited power, because pressure was identical at the rupture point and the sac in 9 of 15 patients. More fundamentally, all outlets were assigned zero relative pressure, a configuration in which the computed luminal pressure is dominated by the inflow-to-outflow drop that drives the prescribed flow; intrasaccular pressure differences of a few hundred pascals then fall below the resolving power of the model. The absence of a pressure difference between the rupture point and the sac should therefore be attributed to this boundary condition rather than read as evidence that pressure is biologically irrelevant to rupture, and our data cannot exclude a genuine role for pressure maxima or local impingement, as others have suggested (13, 14). The discrepancy may reflect the particular pressure metric examined rather than a genuine disagreement; testing the pressure hypothesis would require patient-specific pressure boundary conditions or a pressure metric normalised to the inlet dynamic pressure (13).

### 4.3 Limitations

First, the aneurysm geometries were reconstructed from CTA data, whose finite spatial resolution may affect the accuracy of the reconstructed vessel boundaries, particularly at the aneurysm neck and small branch vessels. Because wall shear stress is sensitive to local vessel diameter and wall curvature, such geometric uncertainty can propagate into the computed TAWSS and OSI values. All models were nonetheless reconstructed using a consistent protocol, which limits systematic bias across the cohort.

Second, the vessel walls were modeled as rigid and impermeable. In reality, cerebral arteries exhibit viscoelastic compliance, and fluid–structure interaction (FSI) studies have demonstrated that wall motion can alter local wall shear stress distributions by approximately 20% relative to rigid-wall simulations, particularly in regions of flow impingement and near the aneurysm neck (20). This simplification may therefore introduce systematic bias in the absolute TAWSS and OSI values reported here, although the relative spatial patterns, low TAWSS and high OSI at rupture points, are less likely to be affected, given that all models share the same rigid-wall assumption. Quantitatively, the reported 20% effect of wall motion on local wall shear stress is smaller than the between-region contrasts we observe, in which median TAWSS differs by approximately a factor of two at each step along the parent artery–sac–rupture point axis. This 20% estimate is, however, sac-averaged; wall compliance acts predominantly in regions of flow impingement and near the aneurysm neck (20), which are not uniformly distributed among the three sampled regions, so the effect cannot be assumed to cancel in the paired contrast between the rupture point and the sac.

Third, a single time–velocity waveform derived from the Doppler ultrasound of one representative patient was applied uniformly as the inflow boundary condition for all 15 cases. This approach neglects inter-patient variability in cardiac output, vascular resistance, and circle of Willis collateralization, all of which influence MCA flow waveforms. Sensitivity analyses have shown that the shape of the inflow waveform can affect time-averaged WSS by 5–15% and OSI by a comparable magnitude (21). Two factors limit the practical impact of this simplification on the present conclusions. Because the design is a within-patient paired comparison, the same waveform drives all three sampled regions of every model, so that waveform-related error is common to the three and cancels in the paired contrasts; and the magnitude of the waveform effect (5–15%) is smaller than the between-region differences reported here (TAWSS differing by roughly a factor of two at each step from parent artery to sac to rupture point). A prospective sensitivity analysis across alternative inflow waveforms was not performed and would further strengthen the argument. The use of patient-specific inflow data would improve the fidelity of individualized hemodynamic assessment. The temporal resolution of the calculations should be considered in the same context: 100 time steps per cardiac cycle is lower than that used in high-fidelity simulations, and OSI has been shown to be the index least robust to the computational solution strategy, with poor agreement between high- and normal-resolution solutions (R^2^ = 0.23) and a systematic underestimation of most indices at lower resolution (22). As discussed in Section 4.2, this resolution error is concentrated in the low-shear regions where directional reversals occur, and its direction cannot be assumed to be conservative; the OSI results are therefore interpreted cautiously.

Fourth, the rupture point was localised by intraoperative visual inspection and therefore represents an anatomical region rather than a geometrically reproducible coordinate; inter-observer agreement was not quantified. Because this uncertainty is of the same order as the spatial scale over which TAWSS and OSI vary within the sac, we did not rank the rupture point by percentile within the sac distribution, as this would imply a spatial precision the localisation cannot support. The analysis was therefore restricted to region-level contrasts. A prospective design combining intraoperative video review by multiple independent observers with image-registered localisation would be needed to allow site-resolved comparison within the sac.

Fifth, the study lacks an unruptured control group. Because only ruptured aneurysms were analysed, it remains unclear whether the observed features (low TAWSS, high OSI) are antecedent risk factors or consequences of rupture. After rupture, intraluminal thrombus formation, flow stagnation, and altered transmural pressure gradients can substantially modify the local hemodynamic environment (31). The low TAWSS and high OSI at rupture points may therefore reflect, at least in part, the hemodynamic consequences of rupture rather than its precursors. Studies including both ruptured and unruptured aneurysms have partially addressed this issue (5, 32), and an analysis of 359 aneurysms characterised the topographic distribution of rupture sites and their relationship to blebs (30), although that analysis resolved the anatomical location of rupture sites without computing site-specific hemodynamics at those points. The within-aneurysm, site-specific design of the present study is a strength; in the absence of a control group, however, these findings should be read as hemodynamic characteristics associated with rupture in time, not as validated predictors of future rupture.

Sixth, the absence of daughter sacs, blebs, and secondary lobulations has two consequences. It removes an important geometric confounder, since a bleb would itself have altered local wall shear stress and its oscillation independently of the rupture (27, 30). It also limits the generalisability of our findings: aneurysms with daughter sacs, which are over-represented among ruptured MCA aneurysms (30), were not represented here, and it remains to be established whether the same hemodynamic signature holds for heavily lobulated lesions, in which local geometry may dominate the shear environment.

## 5 Conclusion

In this CTA-based CFD study of 15 ruptured MCA aneurysms, a within-aneurysm, region-level design showed that the rupture point lay within a locally disturbed, low-shear, high-OSI environment: TAWSS decreased monotonically from the parent artery through the aneurysm sac to the rupture point (all pairwise *p* ≤ 0.003), and OSI was significantly higher at the rupture point than at the aneurysm sac or the parent artery (*p* = 0.0003 and *p* = 0.0002). Because a parent-artery-to-sac-to-dome gradient is a generic feature of saccular geometry, these results are reported as a descriptive regional association rather than as a distinct hemodynamic signature. Luminal pressure, by contrast, did not differ between the rupture point and the sac, indicating that low shear and high oscillatory shear rather than elevated static pressure characterise the rupture point. TAWSS and OSI therefore vary systematically between the rupture region, the aneurysm sac, and the parent artery. Whether such region-level metrics can contribute to rupture-risk assessment requires confirmation in prospective studies. Larger prospective studies with unruptured controls, patient-specific boundary conditions, and fluid–structure interaction are needed to determine whether these parameters precede rupture and can inform clinical decision-making.

## Data Availability

The original contributions presented in the study are included in the article/supplementary material, further inquiries can be directed to the corresponding author.
